# Genome-Wide Association Study of Body Mass Index in a Commercial Landrace × Yorkshire Crossbred Pig Population

**DOI:** 10.3390/vetsci13010084

**Published:** 2026-01-14

**Authors:** Long Jin, Chunyan Bai, Jinghan Chen, Chengyue Feng, Fengyi Dong, Xiaoran Zhang, Junwen Fei, Yu He, Wuyang Liu, Changyi Chen, Boxing Sun, Dali Wang, Hao Sun

**Affiliations:** College of Animal Science, Jilin University, Changchun 130062, China; jinlong25@mails.jlu.edu.cn (L.J.); bcy@mails.jlu.edu.cn (C.B.); 13638887275@163.com (J.C.); cyfeng25@mails.jlu.edu.cn (C.F.); dongfy25@mails.jlu.edu.cn (F.D.); xiaoran24@mails.jlu.edu.cn (X.Z.); feijw22@mails.jlu.edu.cn (J.F.); heyu21@mails.jlu.edu.cn (Y.H.); wuyang24@mails.jlu.edu.cn (W.L.); cychen24@mails.jlu.edu.cn (C.C.); sunpathing@vip.163.com (B.S.)

**Keywords:** GWAS, BMI, pigs, heritability, SNP, candidate genes

## Abstract

The Body Mass Index (BMI), integrating body weight and length, is a widely used metric for obesity assessment in humans. As pigs serve as crucial biomedical models, the application of BMI in swine and its genetic basis remain poorly explored. This study aimed to investigate the genetic architecture of pig BMI and compare two carcass-based BMI metrics (BMI-S and BMI-O) for breeding applicability. A total of 439 Landrace × Yorkshire crossbred pigs were genotyped with a 50 K SNP chip; heritability was estimated via a mixed linear model, and genome-wide association study (GWAS) was performed using the BLINK model. BMI-S and BMI-O exhibited moderate-to-high heritability of 0.55 and 0.47, respectively, with 17 genome-wide significant SNPs detected—including the top associated SNP *rs81382440* on chromosome 4 and *rs80898583* on chromosome 7. Key candidate genes (*GPHN*, *ADAM33*, *KCNH8*, *PDCD4*) and 5 SNP-trait associations validated in PigQTLdb were linked to lipid/energy metabolism and muscle development. Carcass-based BMI improved phenotypic accuracy, and our findings provide core genetic markers and a theoretical basis for molecular breeding of pig body conformation and lipid deposition traits.

## Simple Summary

We analyzed the DNA of 439 Landrace × Yorkshire crossbred commercial pigs and pinpointed specific genetic markers strongly associated with Body Mass Index (BMI). We demonstrated that BMI is a heritable trait and that calculating it based on carcass straight length (CSL) is more reliable for breeding. Several of the implicated genes are known to influence fat metabolism and growth. Our findings enhance the understanding of the genetic architecture governing pig body structure. The identified markers can assist breeders in efficiently selecting animals with superior conformation, promoting healthier herds and potentially improving meat quality, while this work may offer comparative insights into human obesity research.

## 1. Introduction

Pork occupies a pivotal position in global meat consumption, with its yield and quality directly impacting the economic efficiency of animal husbandry and consumer demand [1]. With advancements in breed selection and management practices, consumer preferences have evolved, shifting breeding objectives from a primary focus on growth rate and lean meat percentage towards increased emphasis on meat quality-related traits [2]. Consequently, in-depth research on body conformation and fat deposition patterns in pigs is paramount. Among various metrics, the Body Mass Index (BMI), which incorporates both weight and length information, has garnered increasing attention [3]. However, a primary limitation of BMI is its inability to distinguish between lean muscle mass and adipose tissue, meaning that individuals with different body compositions may possess identical BMI scores. As a composite trait, BMI not only reflects overall growth and development but may also reveal intrinsic links between fat deposition and body structure [4,5].

In human medicine, BMI is extensively used for obesity assessment and metabolic disease prediction, demonstrating its unique value as a composite trait [4,6,7]. Its potential application in pig genetics and breeding is gradually emerging. Individual previous studies have reported that BMI can indirectly indicate the extent of fat deposition in pigs, correlating closely with carcass traits like backfat thickness [8,9], and potentially linking to metrics such as body symmetry [10]. Therefore, BMI serves not only as a comprehensive phenotypic indicator for evaluating slaughter performance and body fat distribution but also provides reference information for meat quality improvement [11]. However, compared to extensively studied slaughter traits like backfat thickness and loin muscle area, research on porcine BMI remains relatively scarce, and its molecular genetic basis has not been systematically elucidated.

The pig Quantitative Trait Locus (QTL) database systematically archives reported functional genetic loci associated with economic traits, providing a crucial reference framework for subsequent GWAS to dissect the genetic mechanisms of complex quantitative traits [12,13]. As a key tool for mining trait-associated genetic variations, GWAS has been widely applied in pig genetics. Numerous studies have used GWAS to analyze various pig populations. These studies identified SNPs associated with three types of traits: growth traits like average daily gain and days to 100 kg, body measurement traits like body length and body height, and backfat thickness. Corresponding candidate genes, such as tribbles pseudokinase 3 (*TRIB3*) and ELOVL fatty acid elongase 6 (*ELOVL6*), were also identified. Among these, Hong et al. also found high phenotypic and genetic correlations between body length and body height traits [14,15,16]. These studies have propelled in-depth exploration of pig growth, development, body conformation, and meat quality traits. However, the BMI indicator is not included in the QTL database, which limits the comprehensive analysis of porcine BMI. The current genetic dissection of economic traits like growth and body measurements in pigs remains incomplete. Association results often fluctuate considerably due to differences in the genetic background of study populations, leading to insufficient consistency across studies. Although the existing QTL database contains numerous records of genetic loci for pig economic traits, the variability in GWAS results across populations makes it difficult to fully validate and supplement the genetic information for some traits, necessitating studies on new populations to refine the related mechanisms. Crossbred populations, owing to their rich genetic diversity, provide excellent material for elucidating the mechanisms underlying complex traits.

The objectives of this study were to determine the heritability of BMI, identify key genomic SNPs and candidate genes, and elucidate its genetic regulatory basis in pigs. We also aimed to lay a theoretical foundation for applying Marker-Assisted Selection (MAS) to improve fat deposition and body conformation. We utilized a population of 439 Landrace × Yorkshire crossbred commercial pigs; using 50 K SNP chip genotyping data and phenotypic measurements of carcass weight and length to calculate BMI, we systematically estimated the genetic parameters of BMI and conducted a GWAS.

## 2. Materials and Methods

### 2.1. Phenotypic Data Preparation

**Figure 1:**
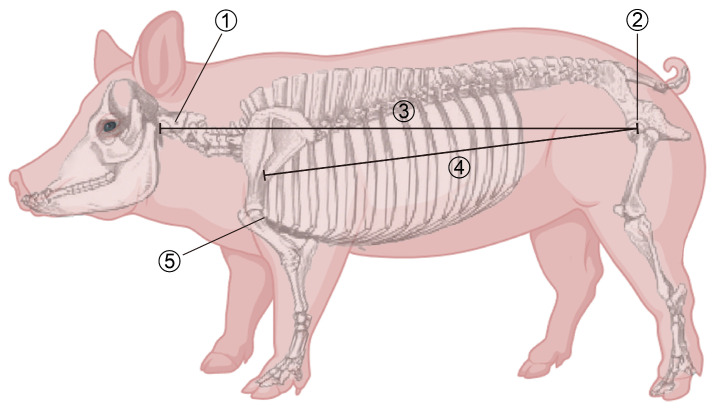
Schematic diagram of measurement positions for CSL and COL. Note: The numbered markers in the figure correspond to the following anatomical structures and measurement items: ① Atlas; ② Pubic symphysis; ③ Carcass straight length; ④ Carcass oblique length; ⑤ First rib. CSL: the straight-line distance from the anterior edge of the pubic symphysis to the anterior edge of the atlas; COL: the straight-line distance from the junction of the first rib and the sternum to the anterior edge of the pubic symphysis.

The experimental animals were sourced from an intensive pig farm in Tongyu County, Baicheng City, Jilin Province, China. The target population was a commercial pig herd derived from rotational crossing of Landrace and Yorkshire pigs. All experimental procedures involving animals were approved by the Institutional Animal Care and Use Committee of Jilin University (Protocol No. SY202507020). These pigs were raised under standardized environmental conditions and feeding regimes to ensure population stability and uniformity. A total of 439 healthy pigs with a uniform age of 180–200 days were selected. All pigs were slaughtered according to industry-standard procedures, during which carcass weight (CW, unit: kg), carcass straight length (CSL, unit: m), and carcass oblique length (COL, unit: m) were accurately recorded for each individual (Figure 1).

Based on the measured CW, CSL, and COL data, the Body Mass Index (BMI) for each pig was calculated using the following formulas:

BMI-S = CW/CSL^2^ (1)

BMI-O = CW/COL^2^ (2)

Here, CSL and COL were used separately to calculate the BMI values for each pig, aiming to compare and determine the more valuable metric for assessing BMI. The significance of the correlation was assessed using a two-tailed *t*-test, with the significance level set at *p* < 0.05.

### 2.2. Genotypic Data Preparation

Following phenotypic data collection, approximately 2 g of fresh muscle tissue was collected from the *Longissimus dorsi* muscle of each pig, placed in centrifuge tubes with 75% ethanol, and stored at −20 °C for DNA extraction. Genomic DNA was extracted using a commercial kit (DC112, Vazyme Biotech, Nanjing, China). Based on silica membrane column purification technology, tissues were lysed with buffer and Proteinase K to release DNA, ethanol was used to bind the DNA to the silica membrane column, and after removing impurities, the purified DNA was isolated. DNA purity was assessed spectrophotometrically using the A260/A280 ratio (acceptable range: 1.8–2.0) and A260/A230 ratio (acceptable range: 2.0–2.2) to evaluate nucleic acid quality. High-quality DNA samples from all 439 pigs were genotyped using the 50 K SNP chip, with genotyping services performed by MolBreeding Biotechnology (Science Park, Hong Kong SAR, China). Initial genotyping results underwent further processing and quality control (QC). Specifically, missing genotype data were imputed using BEAGLE v5.4 (https://faculty.washington.edu/browning/beagle/beagle.html, accessed on 2 July 2025) software to enhance data completeness. Imputed SNP data were subjected to QC using VCFtools (https://vcftools.github.io/index.html, accessed on 3 July 2025) with the following filtering criteria: exclusion of SNPs with a minor allele frequency (MAF) < 1%; exclusion of SNPs with a genotyping call rate < 90%; exclusion of all non-autosomal SNPs. After this QC pipeline, 45,487 qualified autosomal SNPs were retained for subsequent genomic association analyses with BMI traits. The minor allele frequency (MAF) analysis revealed a range of 5.0% to 50.0% (mean = 30.6%) among qualified SNPs, indicating adequate genetic diversity within the genotyped population.

### 2.3. Heritability Estimation

Genetic parameters for BMI were estimated using a mixed linear model implemented in the HIBLUP (V1.6.0) (https://www.hiblup.com/, accessed on 6 July 2025) software. The analysis model was as follows:

y = Xb + Za + e(3)

In the specified model, y denotes the vector of observed BMI phenotypic values. The vector b encompasses fixed effects, specifically age, sex, and slaughter batch. The vector a represents additive genetic effects and is assumed to follow a multivariate normal distribution, a~N (0, Gσₐ^2^), where G is the genomic relationship matrix derived from SNP data to capture genetic relatedness, and σₐ^2^ is the additive genetic variance. The matrices X and Z are the incidence matrices for the fixed and random effects, respectively, e is the vector of residuals, assumed to follow e~N (0, Iσₑ^2^), where I is the identity matrix and σₑ^2^ represents the residual variance. Heritability (h^2^) was calculated as follows:

h^2^ = σₐ^2^/(σₐ^2^ + σₑ^2^) (4)

### 2.4. Genome-Wide Association Analysis

GWAS for BMI was performed using the BLINK model implemented in the GAPIT (V3.0) (https://zzlab.net/GAPIT/, accessed on 6 July 2025) software. Unlike traditional mixed linear models or binning-based methods, this approach iteratively uses two fixed-effect models (FEMs) to select non-highly linked pseudo-QTNs as covariates, based on Bayesian Information Criterion (BIC) and linkage disequilibrium (LD) information. It removes the restrictive assumption of evenly distributed QTNs and avoids REML optimization’s computational cost in random-effect models. This method effectively controls false positives from population structure and cryptic relatedness, while boosting statistical power for causal loci detection and computational efficiency [17]. To mitigate false positives due to population stratification, the first three principal components (PCs) were included as covariates in the analysis. The genome-wide significance threshold was determined by Bonferroni correction, i.e., *p* = 0.05/N, where N is the total number of SNPs. After QC, 45,487 SNPs were retained with a significance threshold of *p* < 1.10 × 10^−6^. SNPs with *p*-values below this threshold were identified as significant loci. Furthermore, the Q-Q plot and genome inflation factor (*λ*) were used to assess systematic bias and population stratification in the GWAS results.

### 2.5. Functional Annotation of Significant Loci

Significantly associated SNPs were mapped to the *Sus scrofa* 11.1 reference genome (Ensembl, https://www.ensembl.org/Sus_scrofa/Info/Index?db=core, accessed on 8 July 2025). The PigQTLdb and PigBiobank databases were queried to determine if these SNPs had been previously reported in association with carcass traits or fat deposition. This provided support for subsequent functional inference. Furthermore, genomic regions 500 kb upstream and downstream of significant SNPs were defined as candidate regions for retrieving protein-coding genes [18,19,20]. This interval was based on the average linkage disequilibrium (LD) decay rate in commercial Landrace × Yorkshire pig populations. It was chosen to conservatively include potential causal variants. Candidate gene annotation primarily relied on the Ensembl database, supplemented by GeneCards and relevant literature for functional characterization.

## 3. Results

### 3.1. Descriptive Statistics of Phenotypes

Table 1 summarizes the descriptive statistics for CW, CSL, COL, and the two calculated BMI types (BMI-S and BMI-O) in the analyzed population of 439 Landrace × Yorkshire crossbred pigs. Analysis of phenotypic variation revealed that carcass weight exhibited the highest degree of variation, with a coefficient of variation (CV) of 9.7%, whereas CSL and COL showed less variation, with CVs of 4.28% and 4.27%, respectively. In contrast, BMI-S and BMI-O, as composite traits, displayed moderate variation (CVs of 7.83% and 7.06%, respectively), indicating that these integrate metrics successfully incorporate the phenotypic differences from both weight and length components.

Phenotypically, BMI-O was significantly higher than BMI-S, a difference directly attributable to the distinct measurement dimensions of CSL (mean 98.49 cm) and COL (mean 84.85 cm). The shorter COL measurement path results in a smaller denominator in the calculation, thereby producing a larger BMI value. Despite this difference in absolute values, the variation trends of the two BMI types were consistent. Both traits exhibited moderate to high heritability, with BMI-S (0.55) being slightly higher than BMI-O (0.47).

### 3.2. GWAS Results

**Figure 2:**
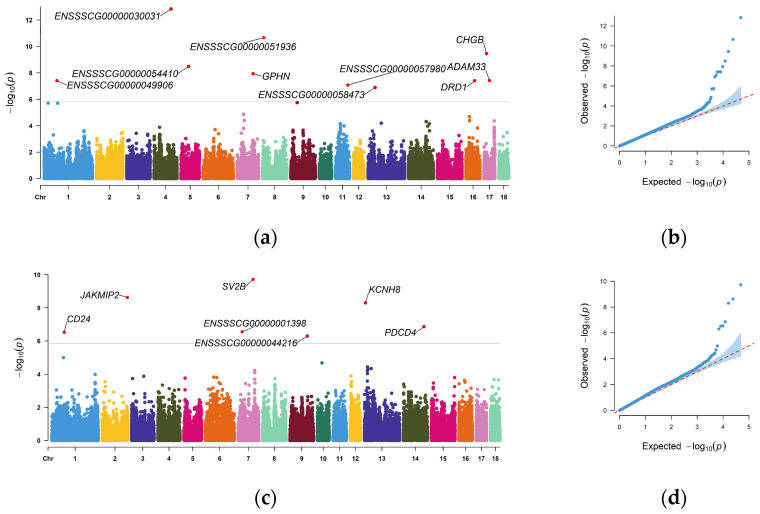
Manhattan and Q-Q plots of genome-wide significant SNPs for BMI-S and BMI-O. (**a**) Manhattan plot for BMI-S, (**b**) Q-Q plot for BMI-S, (**c**) Manhattan plot for BMI-O, (**d**) Q-Q plot for BMI-O.

GWAS was performed on the BMI phenotypes (BMI-S and BMI-O) of the 439 Landrace × Yorkshire crossbred pigs using the BLINK model. The genome-wide significance threshold, determined by Bonferroni correction, was *p* = 0.05/45487, corresponding to −log10(p) = 5.96. Figure 2 presents the Manhattan and Q-Q plots for the BMI-S and BMI-O GWAS results. For both traits, several significant SNP signals deviate from the diagonal in the upper right region of the Q-Q plots, consistent with the signals observed in the Manhattan plots. The genomic inflation factors (λ) were calculated as 1.141 for BMI-S and 1.187 for BMI-O.

Table 2 and Table 3 list the significant SNPs identified for BMI-S and BMI-O traits, respectively. For BMI-S, 10 genome-wide significant SNPs were detected, distributed across nine autosomes: 1, 4, 5, 7, 8, 11, 13, 16, and 17. For BMI-O, 7 genome-wide significant SNPs were identified, spanning six autosomes: 1, 2, 7, 9, 13, and 14. For BMI-S, the top SNP (*rs340169919*, located within the *GPHN* gene) accounted for the maximum phenotypic variation of 11.95%. For BMI-O, the top SNP (*rs81329425*, adjacent to *JAKMIP2*) accounted for 17.25% of phenotypic variation. Among these, 13 SNPs were located in intergenic regions, with distances to the nearest gene ranging from 0.53 kb to 259.57 kb, all within the ±500 kb candidate gene search window.

Among the genome-wide significant SNPs identified for BMI-S, the SNP on chromosome 4 (*rs81382440*) exhibited the strongest association signal (*p* = 1.45 × 10^−13^), with the nearest gene being *ENSSSCG00000030031*. The SNP on chromosome 8 (*rs323983749*, *p* = 2.18 × 10^−11^) and the SNP on chromosome 17 (*rs318768688*, *p* = 3.50 × 10^−10^) had the next strongest association signals after the chromosome 4 SNP (*rs81382440*). For BMI-O, the SNP on chromosome 7 (*rs80898583*) exhibited the strongest association signal (*p* = 1.93 × 10^−10^), with the nearest gene being *SV2B*. The SNP on chromosome 2 (*rs81329425*, *p* = 2.38 × 10^−9^) and the SNP on chromosome 13 (*rs325326100*, *p* = 5.01 × 10^−9^) had the next strongest association signals.

### 3.3. Functional Annotation of Candidate Genes

Among the total 17 significant SNPs, five (*rs81382440*, *rs80821336*, *rs318768688*, *rs81236078*, *rs80818436*) have been previously recorded in the PigQTL database (Release 57), and thirteen are included in the PigBiobank database. Cross-referencing these databases revealed that the significant SNPs from this study are biologically linked to multiple economically important carcass traits, providing potential molecular markers for Marker-Assisted Selection (MAS). Specifically, integration of association results showed that: *rs81382440* is associated with tongue weight, loin muscle area, average daily gain, and lean percentage [21]; *rs80821336* is associated with traits like *Longissimus dorsi* depth [22]; *rs318768688* is associated with traits such as body length, body height, BMI, dressing percentage, average daily gain, and lean percentage [23]; *rs81236078* is associated with dressing percentage, backfat thickness, *Longissimus dorsi* depth, etc. [24]; *rs80818436* is associated with mean corpuscular volume [25]. Consistently, *rs318768688* was also significantly linked to BMI-S (*p* = 3.50 × 10^−10^) in our study. This cross-population confirmation supports its potential as a cross-population core locus for BMI-related breeding. The remaining SNPs are also associated with numerous carcass-related traits: *rs323983749*, *rs81346303*, *rs80898583*, *rs320272214* are associated with lean percentage, backfat thickness, loin muscle area, etc.; *rs322495291*, *rs327543988*, *rs81329425* are associated with loin muscle depth; *rs81445106* is associated with average daily gain and days to 30 kg, etc.; *rs325326100* is not associated with carcass traits but is linked to reproductive indicators like litter size and litter weight. The association of *rs81382440* with growth and meat quality traits supports the existence of a genetic link between porcine BMI and meat quality, consistent with prior studies [26,27], and opens possibilities for indirect meat quality improvement through BMI selection.

We next examined SNPs located within or near known genes. Four SNPs: *rs340169919*, *rs81346303*, *rs325326100*, and *rs320272214* are located within the *GPHN*, *ADAM33*, *KCNH8*, and *PDCD4* genes, respectively. To further clarify the functional roles of candidate genes associated with our significant SNPs, we performed GO and KEGG enrichment analyses for genes within a ±500 kb window (Figures S1 and S2). The GO terms were enriched for processes related to growth and metabolic regulation (e.g., immune response, signal transduction). KEGG pathways included metabolic pathways and the NF-κB signaling pathway, both widely involved in energy metabolism and metabolic homeostasis.

## 4. Discussion

In anthropological and related metabolic health research, body mass index (BMI), as a classic anthropometric parameter, is commonly used to estimate an individual’s fat deposition levels [28]. This study revealed that significant SNPs for BMI traits were distributed across multiple chromosomes, supporting a polygenic regulatory architecture. This finding aligns with the expectation that BMI, as a composite measure of weight and skeletal size, is influenced by numerous synergistic genes and pathways. This is supported by evidence that over 200 genomic loci and core genes like *FTO* and *MC4R* are linked to BMI regulation through interconnected signaling networks [29]. The reliable dissection of such a polygenic trait, however, fundamentally depends on accurate phenotypic measurement. We calculated BMI using post-slaughter carcass traits (CW, CSL, COL) rather than the live body measurements common in earlier research. Live measurements are susceptible to error from animal movement, posture variation, and subjective assessment [30]. In contrast, carcass traits are measured on stationary specimens using standardized anatomical landmarks (Figure 1), which substantially improves phenotypic precision. This methodological refinement is reflected in our heritability estimates, which for BMI-S (0.55) and BMI-O (0.47) were notably higher than those reported in prior studies relying on live-animal data. Johnson et al. [31] estimated the heritability of BMI via the multi-trait REML method in four pig breeds (Landrace, Yorkshire, Duroc, and Hampshire) and reported a moderately low range of 0.16 to 0.25. Wu et al. [32] reported a moderately low BMI heritability of 0.157 in Yunong-black pigs via a single-step GWAS with a 50 K SNP Chip. The enhanced heritability underscores the reliability of our phenotypic data and strengthens the subsequent genetic analyses. In addition, Huang et al. [33] reported that BMI heritability in Dongliao black pigs gradually increases with body weight gain (0.01–0.49). This indicates that fat deposition becomes the main driver of growth in the late stages, so the higher the body weight, the higher the heritability of BMI. Furthermore, we found that BMI-S, which incorporates the torso-reflective CSL, showed a higher coefficient of variation (7.83% vs. 7.06%) and greater heritability than BMI-O, indicating its superior ability to capture genetically regulated variation in body structure and fat deposition. Lastly, most previous heritability estimates for pig BMI have been derived from purebred populations [31,34]. In contrast, our results from a commercial Landrace × Yorkshire crossbred population provide valuable genetic parameter data for hybrids, offering insights into how genetic background influences BMI regulation.

Among the 17 significant SNPs, five and thirteen have been previously annotated in the PigQTL and PigBiobank databases, respectively, where they are linked to various economically important carcass and growth traits. Specifically, four SNPs are located within known genes: *rs340169919* (*GPHN*), *rs81346303* (*ADAM33*), *rs325326100* (*KCNH8*), and *rs320272214* (*PDCD4*). They may influence phenotypic regulation by affecting gene transcription, mRNA splicing, or encoded amino acid sequences. Of these, *GPHN* modulates the *GABAergic* system to affect energy metabolism and body weight in mice [35]. *ADAM33* SNPs are associated with rapid-growth-period body weight in quails [36]. *KCNH8* functions in voltage-gated potassium channels, implying potential roles in growth and metabolism [37,38]. *PDCD4* can promote white adipogenesis by inhibiting the browning of white adipose tissue [39,40]. All four genes are relevant to BMI-related traits. For the intergenic SNPs, the nearest candidate genes include *DRD1*, *CHGB*, *CD24*, *SV2B*, and *JAKMIP2*. Among these, *DRD1* expression can inhibit fat synthesis and promote lipolysis [41]. Pathways associated with *CHGB* involve insulin-like growth factor transport and protein metabolism [42]. *CD24* negatively regulates *PPARγ*, affecting adipocyte differentiation and weight gain in mice [43]. *SV2B* expression correlates positively with indicators of lipid metabolism disorder and body weight [44,45]. The functional enrichment results provide evidence that the genomic regions harboring our significant SNPs are involved in BMI-related biological processes (e.g., fat synthesis, energy metabolism, growth), supporting the hypothesis that these SNPs regulate BMI through such core processes.

In summary, our study offers methodological and practical advancements for porcine breeding. The use of post-slaughter carcass measurements (CW, CSL, COL) to calculate BMI, as opposed to error-prone live measurements, provided highly accurate phenotypic data. Although BMI-O values are inherently higher than BMI-S due to the shorter measurement path of COL, both metrics showed consistent patterns of variation, validating our phenotypic approach. More importantly, BMI calculation relies solely on two easily obtainable metrics, weight and length, offering a low-cost and feasible indirect indicator for preliminary screening of complex traits like fat deposition and body conformation on commercial farms. Notably, this advantage is particularly prominent when compared to the traditional use of B-mode ultrasound to measure backfat thickness as an indicator of obesity. This kind of live measurement is prone to errors because pigs cannot be fully restrained during testing, making them susceptible to environmental stress that causes measurement point deviations [46]. Thus, BMI serves as a more accessible and reliable alternative for obesity-related trait screening in commercial pig breeding, effectively addressing the practical limitations of traditional methods like ultrasound and dissection.

Despite the promising findings, certain practical constraints should be noted. First, validation of the identified markers in diverse independent pig populations is needed. Our study focused on a single crossbred population and slaughter-related phenotypic data are hard to obtain in large quantities. Second, while plausible candidate genes were pinpointed via genomic positioning and functional annotation, their regulatory roles in BMI need further confirmation.

## 5. Conclusions

This study provides the first systematic elucidation of the genetic basis of BMI in crossbred commercial pigs. It also clarifies the breeding advantage of BMI-S and its core associated markers. It not only provides a theoretical basis and practical targets for molecular breeding of “body conformation–fat deposition” in pigs but also provides preliminary genetic information that may be useful for comparative genetic studies between porcine BMI and human obesity. Furthermore, BMI, as an easily measurable phenotype, offers value for the indirect assessment of complex fat deposition traits and can serve as an indirect selection method for complex traits. Future studies may focus on cross-population validation of the identified SNPs, functional characterization of key candidate genes, and their application in commercial pig molecular breeding.

## Figures and Tables

**Table 1:** Descriptive statistics for carcass weight, carcass straight length, carcass oblique length, and body mass index traits in Landrace × Yorkshire crossbred pigs.

Trait	Mean ± SD	Min	Max	CV (%)	h^2^
CW (kg)	83.78 ± 8.12	67.4	99.6	9.7	—
CSL (cm)	98.49 ± 4.22	87	109.3	4.28	—
COL (cm)	84.85 ± 3.62	74.9	98.2	4.27	—
BMI-S (kg/m^2^)	86.39 ± 6.77	68.85	111.04	7.83	0.55
BMI-O (kg/m^2^)	116.31 ± 8.22	85.24	137.07	7.06	0.47

Note: CW = carcass weight; CSL = carcass straight length; COL = carcass oblique length; BMI-S = CSL-based BMI; BMI-O = COL-based BMI. Mean ± SD = mean ± standard deviation; CV = coefficient of variation; h^2^ = heritability. “—” indicates that the heritability was not estimated for this trait.

**Table 2:** Genome-wide association analysis of significant SNPs for the BMI-S trait.

SNP ID	Chr	Pos (bp)	*p* Value	Nearest Gene	Dis (kb)	Variance Explained (%)
*rs81263525*	1	72,860,905	3.94 × 10^−8^	*ENSSSCG00000049906*	9.29	5.37
*rs81382440*	4	97,002,310	1.45 × 10^−13^	*ENSSSCG00000030031*	4.52	10.18
*rs80821336*	5	43,480,338	3.30 × 10^−9^	*ENSSSCG00000054410*	13.96	4.13
*rs340169919*	7	90,911,258	1.15 × 10^−8^	*GPHN*	within	11.95
*rs323983749*	8	7,136,661	2.18 × 10^−11^	*ENSSSCG00000051936*	157.17	10.82
*rs322495291*	11	69,640,052	8.49 × 10^−8^	*ENSSSCG00000057980*	5.61	3.17
*rs81445106*	13	39,747,923	1.28 × 10^−7^	*ENSSSCG00000058473*	2.70	3.63
*rs327543988*	16	49,280,659	3.88 × 10^−8^	*DRD1*	8.87	5.58
*rs318768688*	17	14,734,253	3.50 × 10^−10^	*CHGB*	12.74	7.42
*rs81346303*	17	32,036,473	3.87 × 10^−8^	*ADAM33*	within	2.27

Note: Chr = chromosome; Pos (bp) = position (base pair); Dis = distance; within = SNP located within the gene.

**Table 3:** Genome-wide association analysis of significant SNPs for the BMI-O trait.

SNP ID	Chr	Pos (bp)	*p* Value	Nearest Gene	Dis (kb)	Variance Explained (%)
*rs81236078*	1	73119231	3.00 × 10^−7^	*CD24*	0.53	5.98
*rs81329425*	2	149052527	2.38 × 10^−9^	*JAKMIP2*	6.60	17.25
*rs80818436*	7	23632810	2.78 × 10^−7^	*ENSSSCG00000001398*	1.92	2.13
*rs80898583*	7	87777196	1.93 × 10^−10^	*SV2B*	10.86	6.49
*rs81283839*	9	101894082	5.00 × 10^−7^	*ENSSSCG00000044216*	259.57	3.53
*rs325326100*	13	6149378	5.01 × 10^−9^	*KCNH8*	within	6.65
*rs320272214*	14	121308540	1.38 × 10^−7^	*PDCD4*	within	11.33

Note: Abbreviations are consistent with Table 2.

## Data Availability

The data presented in this study are available on request from the corresponding authors due to the need to ensure standardized and traceable data use, as well as to prevent inappropriate use or unauthorized citation.

## Supplementary Materials

The following supporting information can be downloaded at: https://www.mdpi.com/article/10.3390/vetsci13010084/s1, Figure S1: Gene Ontology (GO) enrichment analysis for candidate genes associated with significant SNPs; Figure S2: Kyoto Encyclopedia of Genes and Genomes (KEGG) pathway enrichment analysis for candidate genes associated with significant SNPs.

## Abbreviations

The following abbreviations are used in this manuscript:

BMI	Body Mass Index
GWAS	Genome-Wide Association Study
SNP	Single Nucleotide Polymorphism
CW	Carcass Weight
CSL	Carcass Straight Length
COL	Carcass Oblique Length
QTL	Quantitative Trait Locus
