# Supplementary figures and images for: Genome-Wide Association Study of Body Mass Index in a Commercial Landrace × Yorkshire Crossbred Pig Population
Source: Vet Sci. 2026 Jan 14;13(1):84. doi: 10.3390/vetsci13010084 (PMC12846655; doi:10.3390/vetsci13010084)

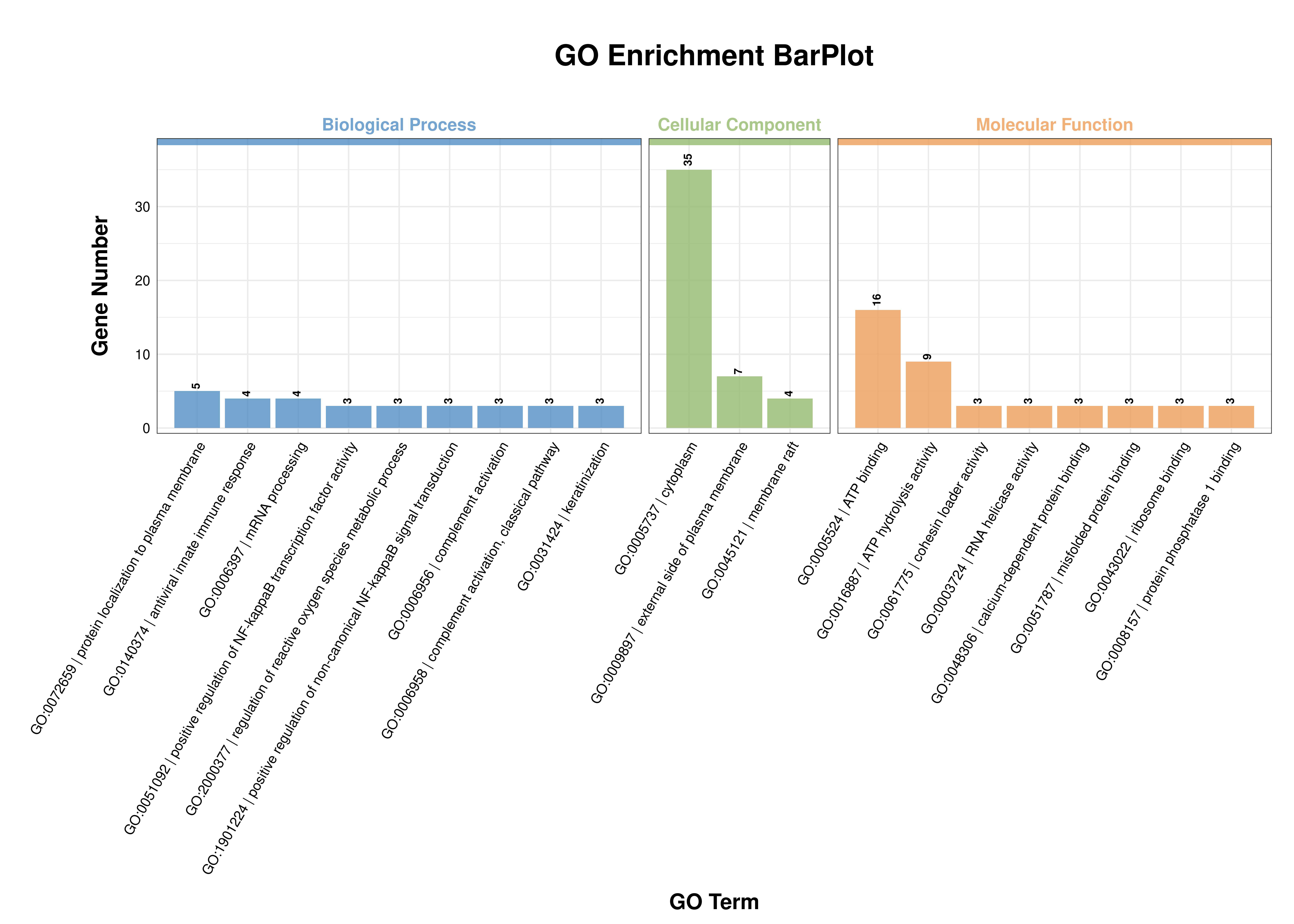

Supplement: Supplementary file 1 [file vetsci-13-00084-s001.zip › Figure S1.png]
